# Outcomes of Head and Neck Neurogenic Tumors

**DOI:** 10.7759/cureus.61156

**Published:** 2024-05-27

**Authors:** Mohamed A Ali, Amr Attia, Iman G Farahat

**Affiliations:** 1 Head and Neck Surgery, National Cancer Institute, Cairo, EGY; 2 Pathology, National Cancer Institute, Cairo, EGY

**Keywords:** malignant peripheral nerve sheath tumor, carotid body tumor, paraganglioma, malignant tumors of neural tissue, schwannomas, neurogenic tumor of head and neck

## Abstract

Background: Lateral neck mass management frequently challenges surgeons. Nerve tissue neoplasms are an uncommon cause of such nodules. Neurogenic tumors form a tiny percentage of the head and neck neoplastic lesions. Considering the number of nerves in this area, it is surprising that such neoplasms are not more frequently seen.

Methods: A retrospective study was conducted on all patients who presented to the National Cancer Institute of Cairo, Egypt, with head and neck neurogenic neoplasms.

Results: During the last 10 years at the National Cancer Institute of Egypt (2006-2015), 40 cases of neurogenic tumors of the head and neck were treated at the head and neck unit. Patients' ages ranged from two to 78 years with a mean age of 34.7 years. Childhood neurogenic tumors accounted for nine cases (22.5%) only in this study. Male patients diagnosed with these tumors comprised 16 cases, while female patients comprised 24 cases, with a female-to-male ratio of 1.5:1. Patient presentation depends on the biological behavior of the tumor; for instance, some of them present by slowly growing painless well-circumscribed mobile swelling, and others present by rapidly growing swelling with neurological deficit. Clinical picture and imaging studies such as CT and MRI raise suspicion and may help delineate such tumors, but a definitive diagnosis is obtained by tissue biopsy. Surgery is the mainstay of treatment in most head and neck neurogenic tumors, whereas adjuvant therapy is of limited benefit in some types of neurogenic tumors. The five-year survival rate was 60% for the malignant group, while death was reported in six out of 15 cases (40%).

Conclusion: Most neurogenic head and neck tumors are benign. Accurate preoperative assessment and a high degree of suspicion are the initial steps in the management. Proper treatment involves complete surgical excision; however, debulking procedures have an important role. Malignant neurogenic tumors are aggressive and are treated with combined radical surgical resection and radiation. Chemotherapy is tried for locally advanced unresectable or metastatic disease.

## Introduction

Neurogenic tumors encompass a tiny portion of all head and neck soft tissue swelling. Managing a solitary mass in the lateral neck is more or less challenging for surgeons. A small percentage of such mass is a neoplasm arising from nerve tissue [1].

Schwann cells, perineurial cells, and fibroblasts are neural crest derivatives. Benign tumors include mucosal neuromas, schwannoma, neurofibroma, dermal nerve sheath myxoma, and granular cell tumors. Malignant tumors include malignant peripheral nerve sheath tumors (MPNSTs) (malignant schwannoma, neurofibrosarcoma), autonomic nerve tumors (plexosarcoma), and malignant melanoma of soft tissues (clear cell sarcoma) [1,2].

Tumors arise from primordial neural crest cells encompassing neuroblastoma, ganglioneuroblastoma, and ganglioneuroma. Neuroblastoma is the least differentiated of these tumors and contains small primitive-appearing cells known as neuroblasts. Paragangliomas are tumors of paraganglionic tissue that contain neuroendocrine tissues. They are a rare neuroendocrine neoplasm. Unlike other types of cancer, no method is identified to determine benign from malignant tumors; therefore long-term follow-up is advised for such tumors [3].

The most important step in the management of such tumors is proper diagnosis, which depends on clinical characteristics, such as the site and onset of a long-standing neck mass. However, these tumors can be incidentally found during scanning for another pathology. Identifying the nerve of origin is a true challenge, as this provides the possibility of informed consent by the patient about any risk of post-surgical resection morbidity. Imaging scans are of low specificity in diagnosing such tumors. The advantage of imaging studies present in treatment planning and evaluation of vascularized tumors; these studies are sometimes very rich but can not diagnose the tumor's nerve of origin, so tissue pathology is important to reach the diagnosis [1].

Treatment strategies depend on the size and site of the tumor, post-surgical functional loss, patient age and profession, and the potential risk of cranial nerve palsy resulting from surgery versus the potential benefit of non-surgical treatment approaches. Generally, younger patients with small tumors in whom no risk or the risk is limited only to one nerve should be treated surgically. On the other hand, older patients who have larger tumors and are at a high risk of injury of multiple nerves and post-operative complications should not be treated surgically. The literature lacks in the determination of the recurrence rate. Recurrence following en-bloc resection is rare. Neurogenic head and neck tumors are radio- and chemo-resistant; radiation therapy should be reserved for palliative treatment in cases where surgery is not advisable [1].

## Materials and methods

Study design

This is a single-institution retrospective study: a retrospective analysis of all the patients who presented to the National Cancer Institute (NCI) of Cairo University, Cairo, Egypt, with neurogenic head and neck tumors cases for surgery from January 2006 until the end of 2015. It retrospectively describes NCI's 10 years of experience with extracranial head and neck neurogenic tumors by presenting their clinical features, diagnostic methods (radiological and biochemical), surgical decisions, and treatment outcomes (functional and oncologic (overall survival and disease-free survival) outcomes).

Study parameters

This study includes patients of all age groups who were diagnosed with extracranial head and neck neurogenic tumors. Data was collected from the biostatistics department. The data included sociodemographic data (age and sex), tumor characteristics (histological type, grade, tumor node metastasis (TNM) staging), surgical complications (post excisional nerve paralysis), and the treatment received (chemotherapy, radiation, or both) and their outcomes. Disease progression was mentioned either as local recurrence and/or distant metastasis.

Statistical analysis

Forty patients were included in this study. Numerical data were identified using means and standard deviations or medians and ranges, as appropriate. Categorical data were summarized as numbers and percentages. Due to the rarity of neurogenic tumors of the head and neck, it was found after consulting medical statistics experts that the best method to describe such rare tumors is to use the methodology of descriptive epidemiology. This method pointed out separately the age, sex, size of the tumor, the presenting symptoms, different methods of management of these cases, and complication outcomes.

Ethical issues

This study is retrospective and poses no harm to patients; all data were anonymous to protect the privacy and confidentiality of patient information.

## Results

In the time between January 2006 and December 2015, 40 cases of head and neck neurogenic tumors at NCI were included in this study. Patients' ages ranged from two to 78 years with an average of 34.7 years. Childhood neurogenic tumors account for only nine cases (22.5%) of this study. Male patients diagnosed with these tumors comprised 16 cases, while female patients comprised 24 cases, with a female-to-male ratio of 1.5:1. The longest dimensions range from 3 to 10 cm, with mean ± standard deviation being 5.4 ± 2.1. The most common presenting symptom was a neck lump and other patient complaints were tumor recurrence or neck pain: neck lump: 20 cases (50%), recurrence: seven cases (17.5%), nasal lesion: six cases (15%), oral cavity mass: three cases (7.5%), skin lesions: two cases (5%), otalgia: one case (2.5%), and epistaxis: one case (2.5%).

Imaging studies

**Figure 1:**
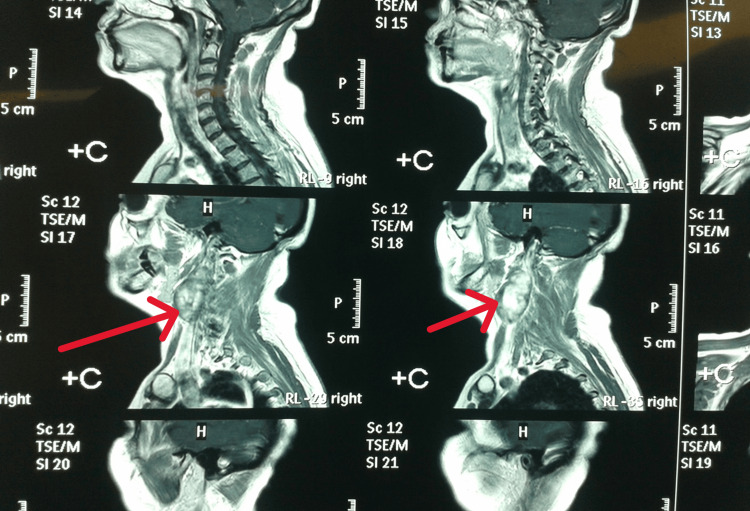
Sagittal MRI section of a cervical schwannoma case

**Figure 2:**
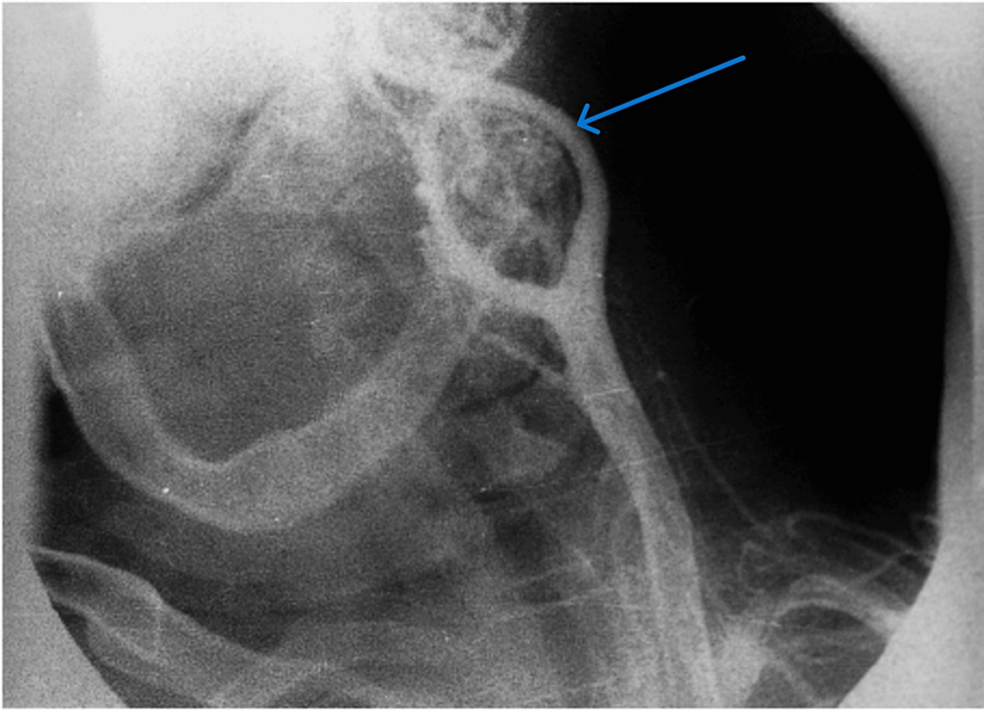
CT angiography of a carotid body tumor (lyre sign) case

The most commonly used investigation was CT, which was used in 17 cases (42.5%), CT angiography was used in nine cases (22.5%), neck ultrasound in four cases (10%), and MRI in four cases (10%), while no imaging study was reported in six cases (15%), especially in skin and oral cavitary lesions (Figures 1, 2).

Pathological pattern

**Figure 3:**
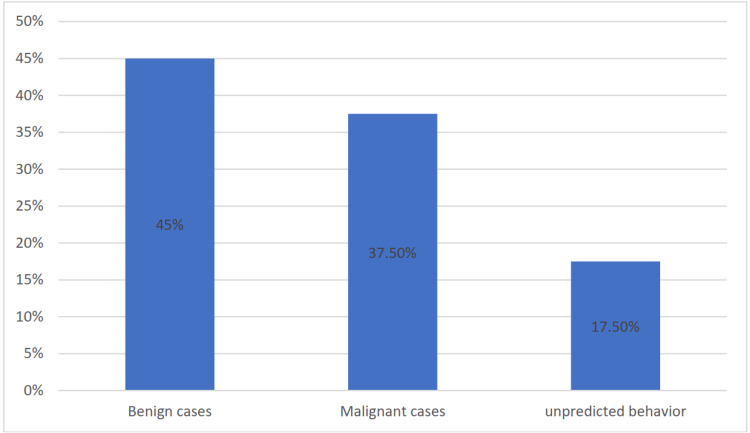
Pathological pattern of the study in the form of percentages

**Figure 4:**
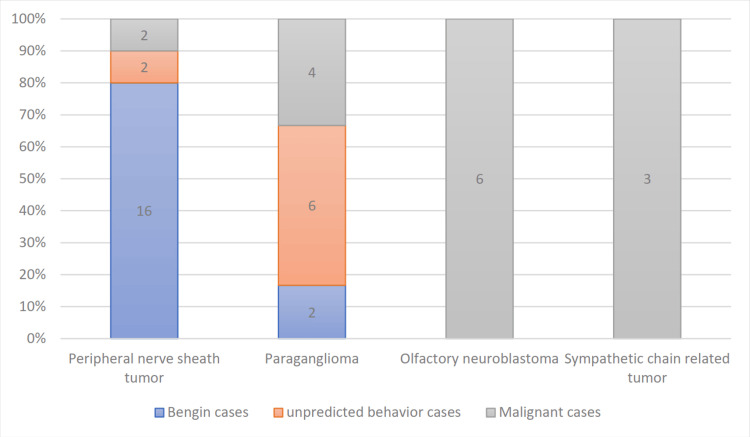
Pathological pattern of each group

Peripheral nerve tumors were the most common neurogenic tumors according to this study, with an incidence of 48%, followed by paraganglioma (30%), olfactory neuroblastoma (15%), and sympathetic chain-related tumors (7%). In this study, benign cases represented 45%, most of them from the peripheral nerve tumor group (Figures 3, 4).

**Figure 5:**
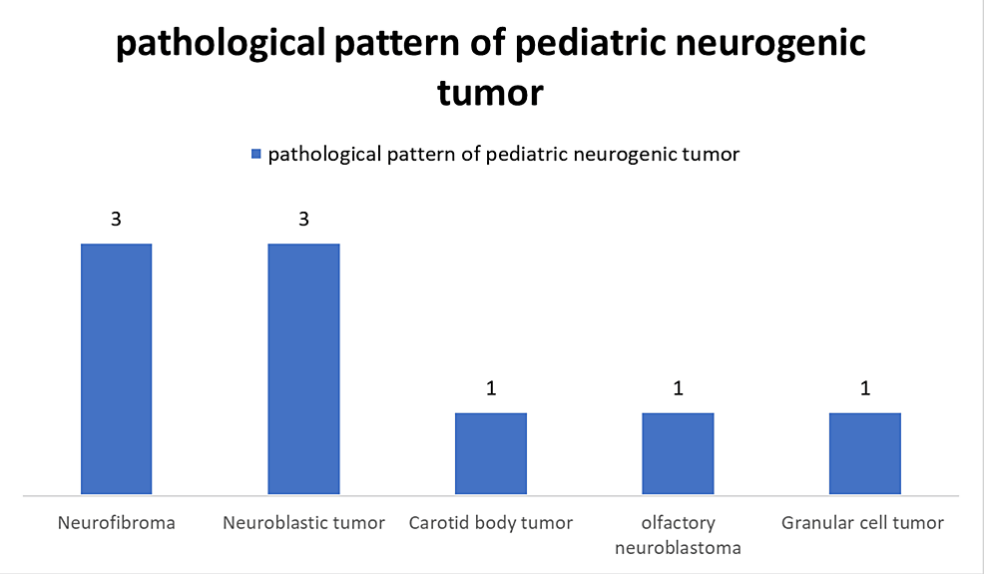
Pathological pattern of pediatric neurogenic tumor

Childhood neurogenic tumors, which accounted for only nine cases (22.5%) in this study, were mostly neuroblastic tumors and neurofibroma (33% for each) (Figure 5).

Treatment and follow-up

Neoadjuvant treatment was received in only seven cases (17.5%), in the form of either chemotherapy (in all cases of olfactory neuroblastoma and one case diagnosed with ganglioneuroblastoma) or combined chemoradiotherapy (CCRTH) in one MPNST case with intracranial extension (T4N3).

Surgical management was the mainstay of treatment in this study, which was carried out in all except four cases, where the patients died when they were receiving their neoadjuvant treatment. The type of surgery was wide local excision in 20 cases (50%), wide local excision and lymph node dissection in all paraganglioma cases (27.5%) except one metastatic case at the time of presentation, minimally invasive surgery (7.5%) in three out six cases of olfactory neuroblastoma and partial maxillectomy through a transfacial approach in one olfactory neuroblastoma case. The most common sites of metastatic spread were lung, bone, and cervical lymph nodes in five cases, four of them due to malignant carotid body tumors (CBTs), and one was due to an olfactory neuroblastoma case.

Post-operative adjuvant treatment was received in eight cases: radiation treatment was received in two CBT cases metastatic to bone and cervical lymph node, one olfactory neuroblastoma case metastatic to cervical lymph node, and one MPNST with positive margins. Chemotherapy treatment was used in neuroblastic tumor cases and one CBT case metastatic to the lung, while chemoradiation treatment was received in one CBT case metastatic to the lung and bone. 

Outcomes

**Figure 6:**
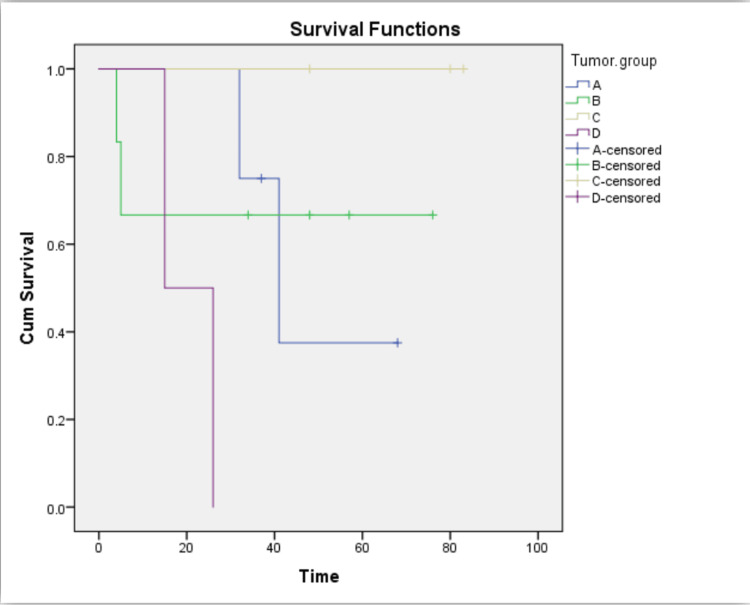
Overall survival

From the whole sample, complications had occurred in 13 cases (32.5%), most of them during paraganglioma dissection (eight cases). Vascular injuries were the most common complication with a percentage of 46% out of all complications. They were found in six cases, most of them due to external carotid artery ligation, while other complications included cranial nerve injury in two cases and combined injuries in two cases. Speech defect occurred in wide local excision of tongue granular cell tumor. Due to the tumor's local extension, Horner's syndrome and quadriplegia were reported in two cases, which were diagnosed as neuroblastoma and MPNST. Mortality was reported in six cases out of 15 cases of the malignant group (40%): malignant paraganglioma group - two cases out of four (50%), olfactory neuroblastoma group - two cases out of six (33.3%), and MPNST group - two cases out of two (100%). Statistical analysis for survival was done using the Kaplan-Meier method (A for the malignant CBT group, B for the olfactory neuroblastoma group, C for the neuroblastic tumor group, and D for the MPNST group) (Figure 6).

## Discussion

In this study, 40 patients with benign and malignant neurogenic tumors arising in the head and neck region are described. The macroscopic and histologic characteristics of these tumors were related to their prognosis and treatment. The nerve sheath tumors present in this region were similar to those that arise anywhere else. Neoplasms of sympathetic nervous system origin were uncommon; only three neuroblastomas were seen. Possible difficulties in the microscopic interpretation of olfactory neuroblastomas were present. In many of these patients, there was a surprising lack of correlation between their clinical course and the microscopic characteristics of their neoplasm.

**Figure 7:**
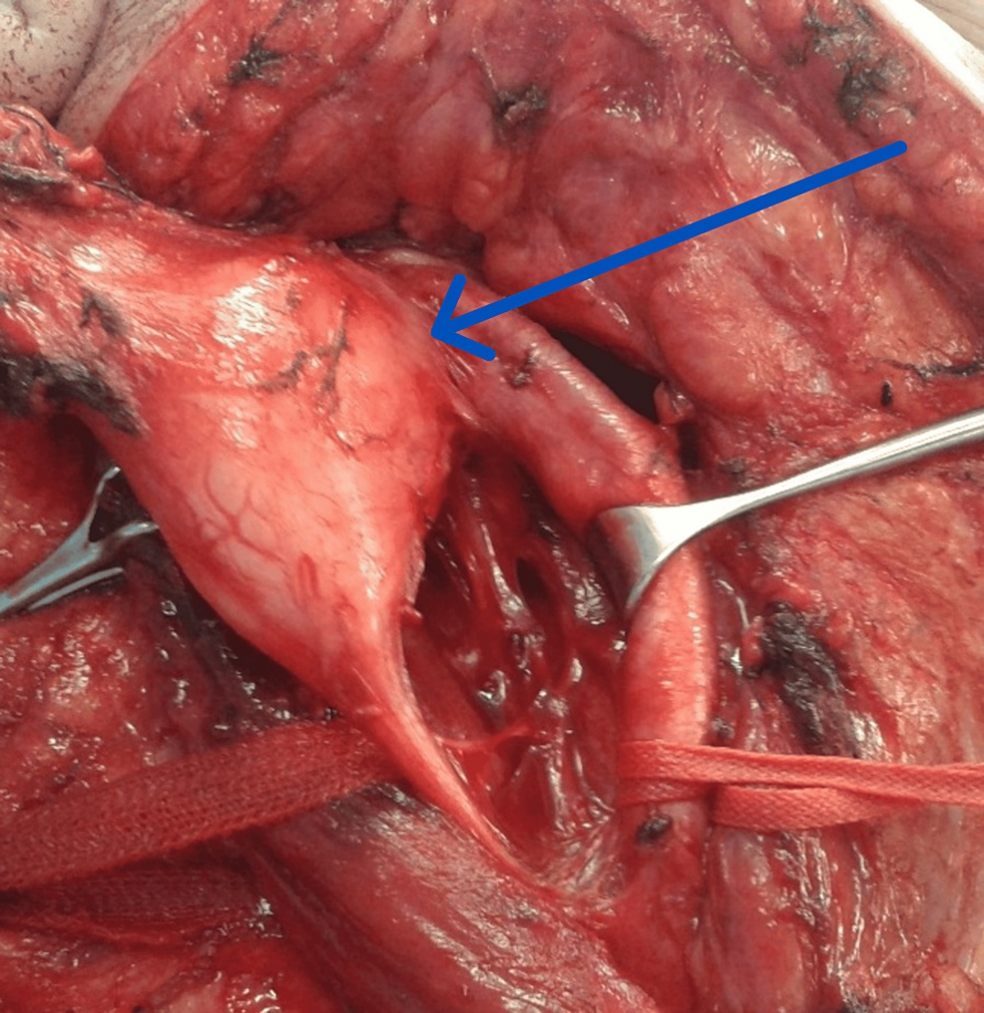
Intraoperative finding of vagal schwannoma in one of the cases

The majority of head and neck neurogenic tumors are benign. The first line in the management involves accurate preoperative evaluation and a high index of suspicion. The principle of treatment involves complete surgical resection, R0 resection, but debulking surgical excision has a definite role. Malignant nerve sheath tumors are aggressive and are treated with radical surgical debulking followed by radiation. Unresectable or metastatic diseases are best treated by chemotherapy. Peripheral nerve tumors were the most common neurogenic tumors according to this study, with an incidence of 48%, followed by paraganglioma (30%), olfactory neuroblastoma (15%), and sympathetic chain-related tumors (7%). In our study, the most common presentation was a painless neck lump, one case presented to NCI as a tongue squamous cell carcinoma; diagnostic difficulties may arise because histopathological features of the pronounced pseudoepitheliomatous hyperplasia can be mistaken with a well-differentiated oral squamous cell carcinoma. Definitive diagnosis is achieved only by tissue biopsy. The age of patients with peripheral nerve tumors in the head and neck was found to be from two to 78 years in the patients of this current study with a mean age of 30 years, which is slightly younger than the mean age in the literature review [4,5]. There was a slight female predominance in this study while there was no sex predilection in the literature review. The average diameter was 5 cm, which is the same size as in the literature review. The most encountered tumor was schwannoma, then neurofibroma and granular cell tumor (Figure 7).

Regarding MPNSTs, Loree et al. mentioned that local recurrence is related to tumor size and negative margin achievement. No local recurrences occurred in those patients who had negative margins of resection and received adjuvant radiotherapy. Tumor grade was predictive of the development of distant metastases. Resection with negative margins is the principle of the local control concept; adjuvant radiotherapy may help in this group. Salvage surgery may be of benefit in local recurrence [4]. In this study, the incidence of malignant peripheral nerve tumors was 10%; most tumors were irresectable due to local extension at the time of diagnosis, so when the tumor was resectable, a multi-modality approach involved surgical debulking and then postoperative radiation especially if margins were positive. However, the recurrence rate was 100 %. The median overall survival in this study was 20.5 months, while Wanebo et al. found that the median overall survival time was 44 months. Overall survival was significantly influenced by patient age, tumor location, tumor size, extent of surgery, and quality of margins. Patients with a family history of neurofibromatosis also had better disease‐free survival [5].

Concerning paraganglioma, according to Luna-Ortiz et al., the age of the patients ranged from 18 to 94 (mean = 50.2). The most common chief complaint was a painless neck mass (78.7%). Women significantly predominated (96.9%) with a female: male ratio of 31.2:1. All patients except one were evaluated with a preoperative angiography [6]. In this study, patient ages ranged from 18 to 67 years, with an average of 37.1 years, which is slightly younger; there was also female predominance (66.7%) in this study. The average size of this tumor was 5.8 cm, ranging from 3 to 10 cm, which is similar to those in the literature review [7]. CT angiography was the most common imaging study used for preoperative evaluation of such tumors (67%), followed by CT and MRI. To avoid costly mistakes, one must ensure the whole differential diagnosis of the cervical mass during the initial evaluations. Quite similarly, between the presentation of CBT and jugulodigastric lymph node, metastasis is reported.

**Figure 8:**
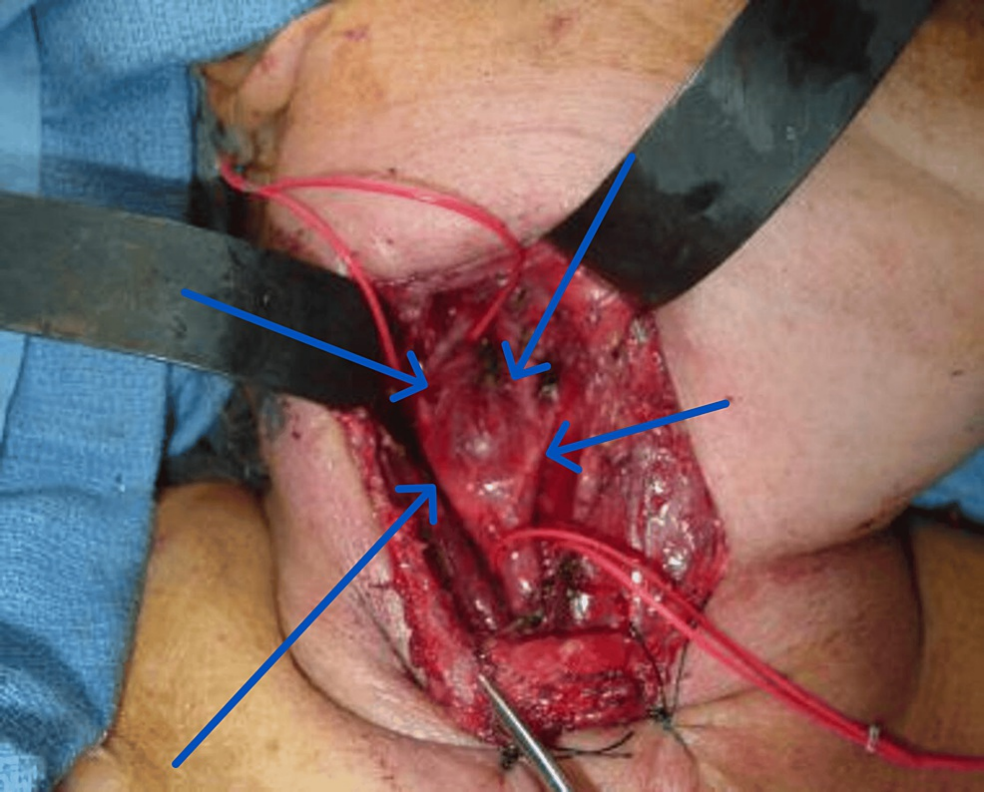
Intraoperative finding of carotid body tumor splaying carotid bifurcation in one of our cases

The main line of treatment was surgical removal in patients of our study (92%), and only one patient who presented with recurrence and chest deposits received chemotherapy. The main line of treatment according to Frijns et al. was surgical management: the most used approach was transcervical. One patient, with tumor recurrence, in this study required the skull base technique. Wide exposure was a must for meticulous hemostasis. Early control of the proximal and distal vessels was then accomplished with vessel loops. Important neurovascular bundles were identified and appropriately retracted and reserved. The tumor was then carefully dissected from the common carotid. This dissection was carried out superiorly, making every effort to protect the internal carotid artery. If necessary, the external carotid was ligated. [7]. Conservative methods of treatment such as the wait-and-see policy were not followed in this study, which may be due to a lack of patient compliance. Early surgical management is recommended to avoid neurological deficits. However, Frijns et al., reported that for skull base and bilateral glomus tumors, a more conservative monitored wait-and-see policy can be sensible and should be considered in any proposal for the treatment of head and neck paragangliomas. However, surgical intervention is highly recommended if intracranial progression or serious affection of cranial nerves happens, such as nerve palsy, to reduce morbidity rather than trying to increase survival rates (Figure 8) [7].

The incidence of benign paraganglioma in this study was 16.7%, unpredicted behavior tumor was 50%, and malignant nature was 33.3%. The most common sites of metastasis were lung, bone, and cervical lymph nodes. In concordance with this study, Nishijima et al. stated that most CBTs are benign; however, some can show malignant behavior [8]. Malignant CBTs have an unpredictable history; often, there is no correlation between the histologic findings and the clinical behavior. They are usually diagnosed by the development of local recurrence or lymph node metastasis following total resection of the primary mass, or by the detection of distant metastasis.

In a metanalysis by Amato et al. to review surgical outcomes, they found that total cranial nerve injuries were 48.32%, of which 31.04% were transient and 1.28% were permanent; arterial injuries were 27.84%, most of which were external carotid artery (ECA) injuries. Cerebrovascular accidents due to surgery were 2.4%. They concluded that surgical resection remained the treatment of choice for these diseases despite the related morbidity [9]. In this study, vascular injuries were the most common complication (46%), mostly due to external carotid artery (ECA) injuries and common carotid artery injuries. Shunt was reported in two cases and cranial nerve injuries were 25% out of all complications. To achieve no or low surgical complications with these resections, high cervical exposure with care should be done, following the identification of all the cranial nerves before tumor resection.

**Figure 9:**
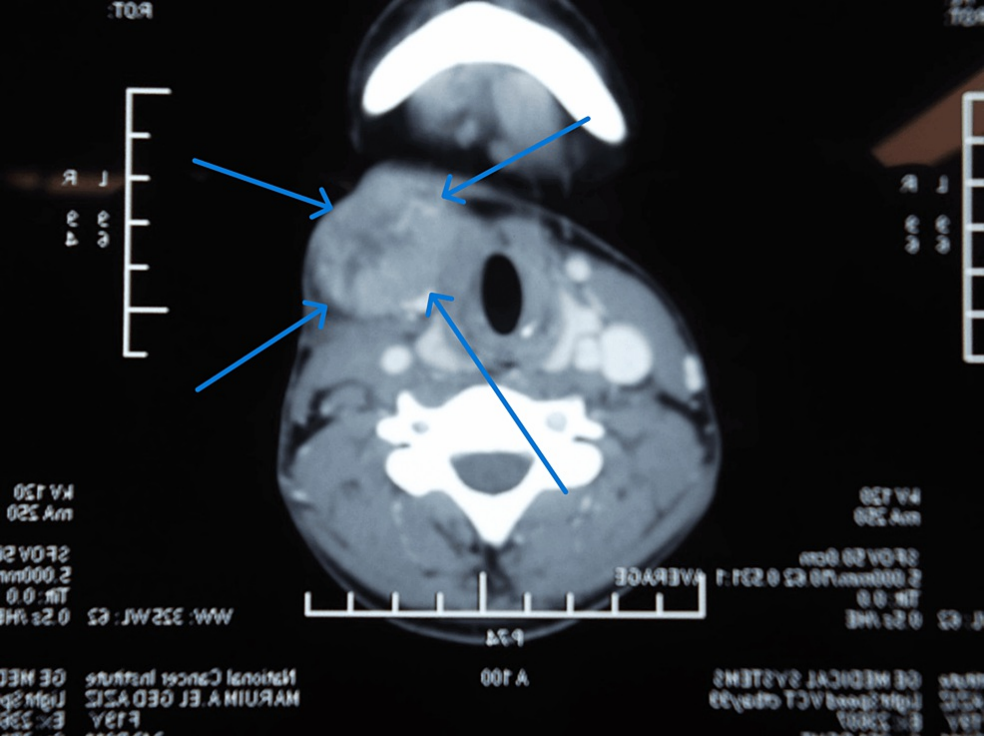
CT scan with contrast of one of our cases with recurrent carotid body tumor

The overall survival rate according to Kotelis et al. was 82.4%; the disease-specific survival rate was 94.1% (16 of 17 patients). One patient (5.6%) died of local tumor recurrence three years after an R1 resection [10]. In our study, when malignancy was proven, the overall survival was 45 months and disease-free survival was 33 months, recurrence occurred in three cases (75.5%), and death was reported in two cases (50%). Locoregional control is usually obtained with complete primary tumor resection lymphadenectomy and eventual radiotherapy. Surgery followed by radiation therapy on the isolated metastatic sites seems to be effective. Current multidisciplinary treatments failed to control the gross metastatic disease (Figure 9).

Concerning olfactory neuroblastoma, the clinicopathologic features of olfactory neuroblastomas reviewed by Mills and Frierson showed that in patients with ages ranging from nine to 66 years (median: 49 years), 57% were women; six patients (29%) developed local recurrences and eight cases (38%) had metastases. Ten patients (48%) were alive and free of disease from 10 to 239 months (median: 58 months) after diagnosis. Seven (33%) died of the tumor, with a duration of survival ranging from one to 142 months (median: 27 months) [11]. In this study, patients' ages ranged from 4 to 73 years and the mean was 45 years, with a male-to-female ratio of 1:1. The most common presenting symptoms were nasal mass and epistaxis. Four patients (67%) were disease-free from 34 to 76 months (median: 107.5 months) after diagnosis. Two patients (33%) died of the tumor and survived only from four to five months while receiving chemoradiation therapy.

**Figure 10:**
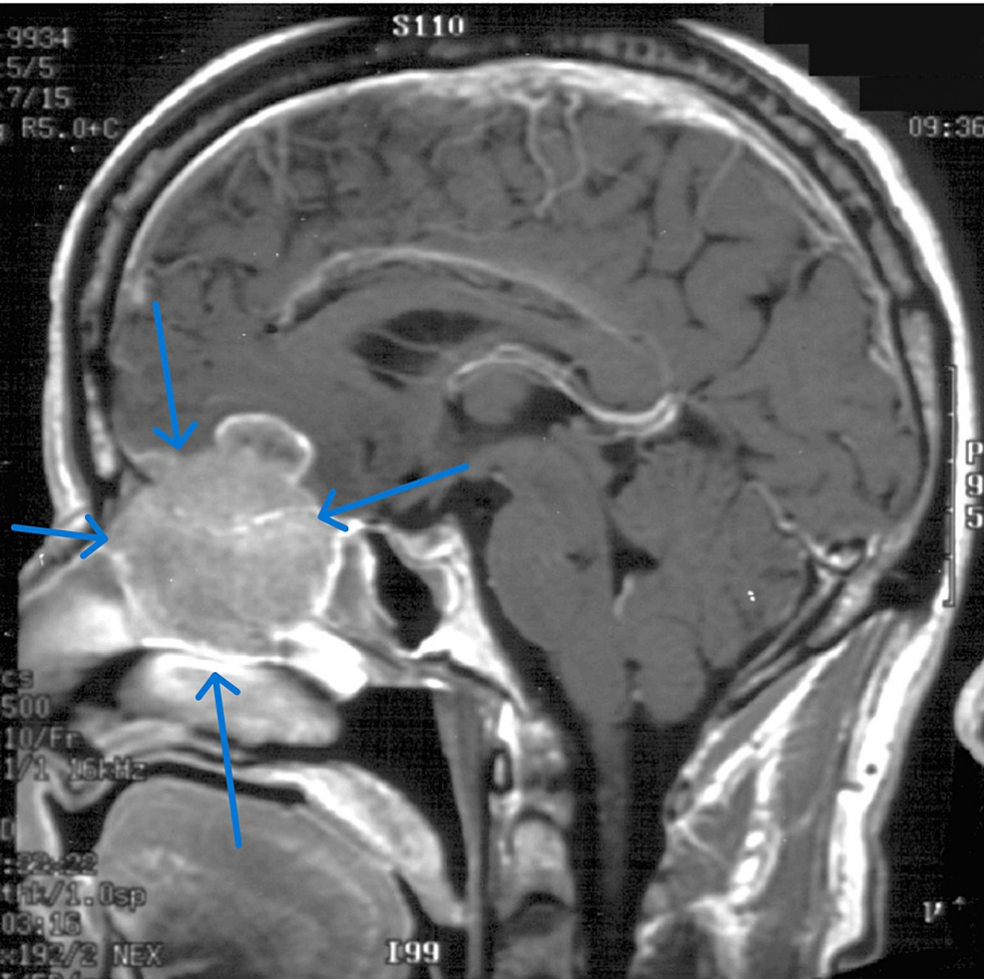
MRI sagittal section scan with contrast of olfactory neuroblastoma extends cephalad to anterior cranial fossa through the cribriform plate

In our study, management in all cases was neoadjuvant CCRTH first then surgical excision. Cervical lymph node recurrence occurred in one case after 56 months with an incidence of 16%, treated by radical neck dissection and then radiotherapy. Also, bony metastasis was reported in that case and the patient received radiotherapy. Minimal invasive surgery such as endoscopic sinus surgery was superior to the transfacial approach with incidences of 75% and 15%, respectively. The findings in the present study have important indications for endoscopic techniques in the future. Endoscopic resection of small tumors followed by radiotherapy may give comparable results; however, a great effort must be taken in terms of case selection and long‐term follow‐up if similar results to the craniofacial in terms of survival and morbidity are to be obtained; given the difficulty in the proper diagnosis of tumor extensions, surgeons must be prepared and trained in craniofacial resection if the endoscopic approach fails (Figure 10).

The role of chemotherapy in the literature review, either before or after RT or surgery, is unclear [11]. Two cases in this study died while receiving neoadjuvant CCRTH. However, one study by Kim et al. showed that nine out of eleven patients achieved objective responses (objective response rate: 82%; 95% confidence interval: 52-95%), which included two complete responses and seven partial responses. The major side effect was hematologic toxicity such as neutropenia. All chemotherapy complications were reversible, and no chemotherapy‐related mortality was documented. The median survival period was 18 months (range three-45 months).

Concerning sympathetic chain-related tumors, Moukheiber et al. described four pediatric patients with head and neck neuroblastic tumors. Ganglioneuroblastoma was in three cases and neuroblastoma in one case. Presenting pictures were a single cervical mass in two cases and respiratory distress in association with Horner's syndrome in two cases. The mean age at presentation was 15 months. High levels of urine catecholamine were seen only in the patients with neuroblastoma. Surgical treatment was performed on all patients. Neoadjuvant chemotherapy was performed in one case. No evidence of recurrence was observed with a mean follow-up period of seven years [12].

**Figure 11:**
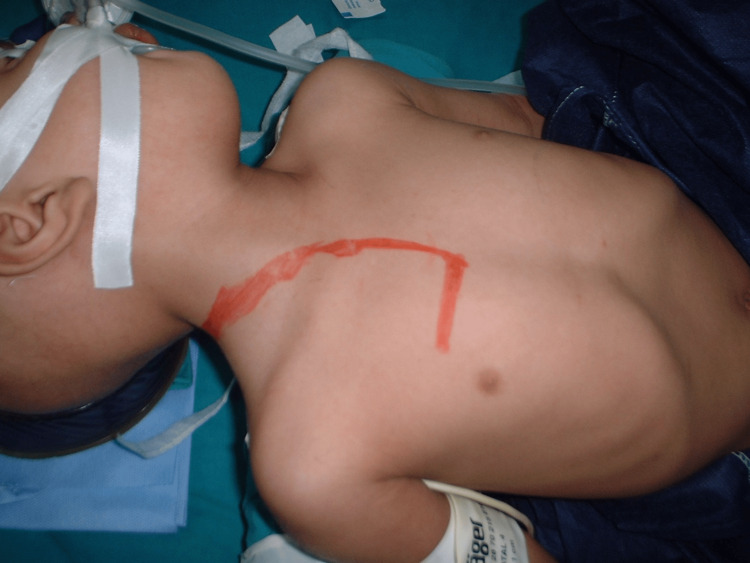
Trap door incision in one of our cases where the patient presented with cervical neuroblastoma

**Figure 12:**
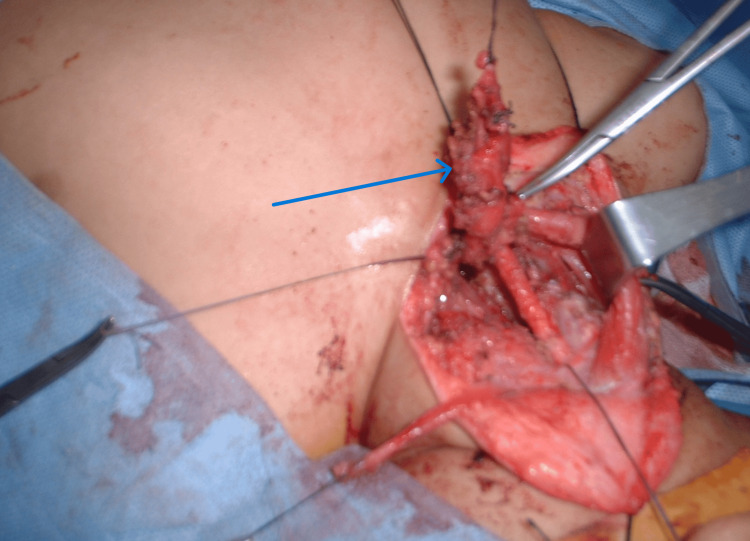
Trap door incision in one of our cases where the patient presented with cervical neuroblastoma

In our study, the first decade of life was a common pool of incidence for this tumor. The female-to-male ratio was 2:1 in this study, while it's equal in both sexes in the literature review [12]. The presenting symptoms (neck swelling, Horner's syndrome) in the patients of this study with increased urine catecholamine levels were observed only in the patients with neuroblastoma. Only three cases were diagnosed with such tumors in this study with an average size of 5 cm. Neoadjuvant chemotherapy was performed in ganglioneuroblastoma. Surgical-wide local excision was the main line of treatment followed by adjuvant chemotherapy in ganglioneuroblastoma and neuroblastoma cases only with tumor regression. The overall survival ranged from 48 to 83 months, with a mean survival of 70.3 months (Figures 11, 12).

Primary neuroblastoma of the head and neck is rare. The clinical presentation and physical findings are related to the child's age, the stage and location of the primary tumor, and its attendant biological behavior. The prognosis of these lesions has been increasingly favorable. Carefully planned surgical approaches provide local control, and radiation and chemotherapy may be beneficial in more extensive tumors.

Study limitations

Due to the retrospective character of this study, some limitations need to be considered when interpreting the results. The most important one is the rarity of such tumors, which is reflected in the statistics. Furthermore, the difficulty of pathologic diagnosis of neurogenic tumors is emphasized by the number of neoplasms excluded from this series after a critical review of clinical and microscopic findings.

## Conclusions

The patients' mean age was 34.7 years. Childhood neurogenic tumors account for only 22.5% in this study, with a female-to-male ratio of 1.5:1. The presenting complaints of such patients depend on the biological behavior of the tumor. Some of them present with a slowly growing painless well-circumscribed mobile lump, and others present with a rapidly growing swelling in addition to neurological symptoms.

Most neural-derived tumors in the head and neck region are benign. Accurate preoperative evaluation and a high degree of suspicion are important parts of the management, but a definitive diagnosis is obtained by tissue biopsy. Complete resection could be achievable and is considered a treatment that can cure the tumor; however, debulking procedures have a definite role. Resection and then reconstruction of the affected nerve should be attempted if serious affection happens. MPNSTs are the most aggressive and are treated with radical surgical resection followed by radiation whenever possible. Chemotherapy may be tried as a palliative treatment for unresectable or metastatic disease.
